# *Hermetia illucens* and *Hermetia fenestrata* (Diptera: Stratiomyidae) Colonization of “Spoiled” Stingless Bee *Geniotrigona thoracica* (Hymenoptera: Apidae) Hives in Malaysia

**DOI:** 10.3390/insects11110737

**Published:** 2020-10-27

**Authors:** Tania Ivorra, Martin Hauser, Van Lun Low, Jeffery K. Tomberlin, Natasha Azmi Nur Aliah, Jonathan A. Cammack, Chong Chin Heo

**Affiliations:** 1 Department of Medical Microbiology and Parasitology, Faculty of Medicine, Sungai Buloh Campus, Universiti Teknologi MARA, Sungai Buloh 47000, Selangor, Malaysia; aliahazmi38@gmail.com (N.A.N.A.); chongchin83@yahoo.com (C.C.H.); 2 Plant Pest Diagnostics Branch, California Department of Food & Agriculture, 3294 Meadowview Road, Sacramento, CA 95832-1448, USA; martin.hauser@cdfa.ca.gov; 3 Tropical Infectious Diseases Research and Education Centre (TIDREC), University of Malaya, Kuala Lumpur 50603, Malaysia; vanlun_low@um.edu.my; 4 Department of Entomology, College Station, Texas A&M University, TX 77843, USA; jktomberlin@tamu.edu; 5 EVO Conversion Systems, LLC, 5552 Raymond Stotzer Pkwy, Suite 100, College Station, TX 77485, USA; jacammack@evoconsys.com; 6 Laboratory and Forensic Medicine (I-PPerForM), Institute for Pathology, Sungai Buloh Campus, Universiti Teknologi MARA, Sungai Buloh 47000, Selangor, Malaysia

**Keywords:** stingless bee, Apidae, soldier flies, *Hermetia*, Malaysia

## Abstract

**Simple Summary:**

Since 2012, the stingless beekeeping industry in Malaysia has been booming and to date, there are more than 700 farms representing around 60,000 colonies. In 2019, a group of fly immatures were found in a decaying stingless bee nest. The species identification for adults and larvae was carried out by both morphology and molecular techniques. This study provides the first identified record of *Hermetia fenestrata* colonizing a “spoiled” stingless bee colony. The implementation of proper management of stingless bee farms and novel strategies in the prevention and control of *Hermetia* colonization should be further explored in order to maintain healthy bee colonies for mitigating losses.

**Abstract:**

Meliponiculture, the keeping of domesticated stingless bees such as *Geniotrigona thoracica* (Smith, 1857) (Hymenoptera: Apidae), is an increasingly popular agricultural industry in Malaysia. This study reports the soldier fly (Diptera: Stratiomyidae) species of the genus *Hermetia* colonizing stingless bee colonies in Malaysia. The larvae were reared in the laboratory to the adult stage and identified through molecular and morphological approaches. *Hermetia illucens* (Linnaeus, 1758) and *Hermetia fenestrata* de Meijere, 1904 (Diptera: Stratiomyidae) were identified from the sample provided. Earlier records of stratiomyids in stingless bee nests were misidentified as *H. illucens*. This paper represents the first identified record of *H. fenestrata* colonizing a “spoiled” stingless bee colony. In addition, adult and larval morphological differences between both species and the roles of both species in bee nest decomposition are discussed.

## 1. Introduction

Meliponiculture is the keeping of stingless bees (Hymenoptera: Apidae), in which a colony of bees is domesticated for the purpose of pollination or for the production of honey, pollen, and propolis products [1]. A healthy stingless bee colony has about 5000 worker bees, housed inside a wooden hive box and includes propolis pots that store honey and pollen. These hives should be monitored daily by checking the number of bees flying in and out of the box (a strong healthy hive has more than 30 bees per minute). However, a spoiled hive usually is a weak hive with less than 10 bees flying in and out per minute and where the propolis pot is ruptured and the honey and pollen are spilled into the box. Modern beekeeping in Malaysia began during the early 1980s with local bees *Apis cerana* Fabricius, 1793 (Hymenoptera: Apidae) and the importation of commercial bee species, such as the European *Apis mellifera* Linnaeus, 1758 (Hymenoptera: Apidae), which were capable of producing 50 kg of honey per colony annually. However, these species were determined to be susceptible to select pathogens [2,3]. In 2004, the Malaysian Agricultural Research and Development Institute (MARDI) introduced several species of stingless bees in order to avoid infectious diseases. Since 2012, the stingless beekeeping industry in Malaysia has been booming [2,4,5].

Stingless bees, also known as *lebah kelulut* in Malay language, are common and well adapted to tropical and subtropical regions of the world, such as Central and South America, Africa, Northern Australia, and Southeast Asia [5,6]. They belong to the tribe Meliponini, which collect and store nectar to produce honey. Their main characteristic is their lack of functional stingers [1,6,7]. In Malaysia, nearly 40 different species are known to be native [8]; however, meliponiculture is restricted to only two native domesticated species: *Geniotrigona thoracica* (Smith, 1857) and *Heterotrigona itama* (Cockerell, 1918) (Hymenoptera: Apidae) [1,3,4,5,6,9,10].

*Geniotrigona thoracica* is one of the largest Malaysian stingless bees (~8.39 mm long) [1,11] with a yield of approximately 2–5 kg of honey per colony annually [2,6]. Recently, total beekeepers in Malaysia numbered more than 700 farms representing 58,593 colonies [2,5,6]. However, in 2016, a large number of colonies declined, resulting in losses of over 1 million Malaysia Ringgit (equivalent to ~USD 250,000). In some of these colonies, Hashim et al. [12] found large numbers of stratiomyid larvae and were considered to be the cause for the decline of the colonies. However, two major flaws were found in the Hashim et al. [12] study: First, the species was misidentified as *H. illucens* [13], and second, there was no clear evidence (e.g., feeding or predation activities on bee larvae) demonstrating *H. illucens* was the causative agent leading healthy bee colonies to collapse. Since then, it has been debated if *H. illucens* was actually the culprit colonizing stingless bees. For this reason, the objective of the current paper was to confirm the stratiomyid species infesting stingless bee colonies.

## 2. Materials and Methods

**Figure 1:**
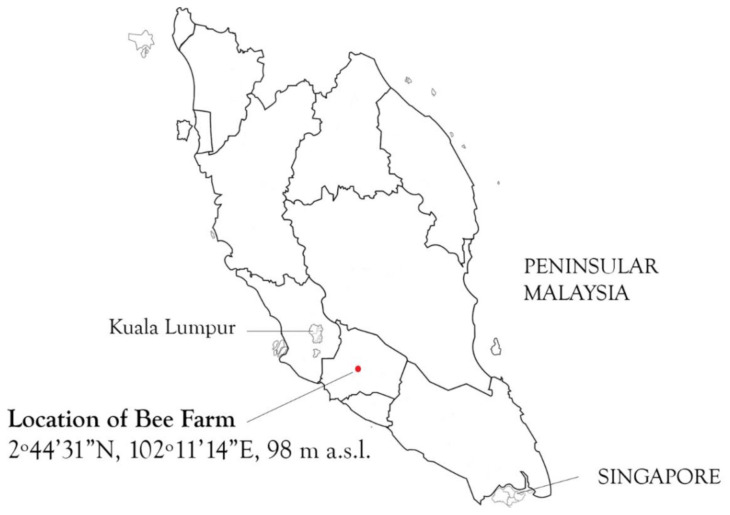
Map of peninsular Malaysia showing the location of the bee farm where *Hermetia illucens* and *H. fenestrata* (Diptera: Stratiomyidae) were found colonizing *Geniotrigona thoracica* (Hymenoptera: Apidae) nests.

**Figure 2:**
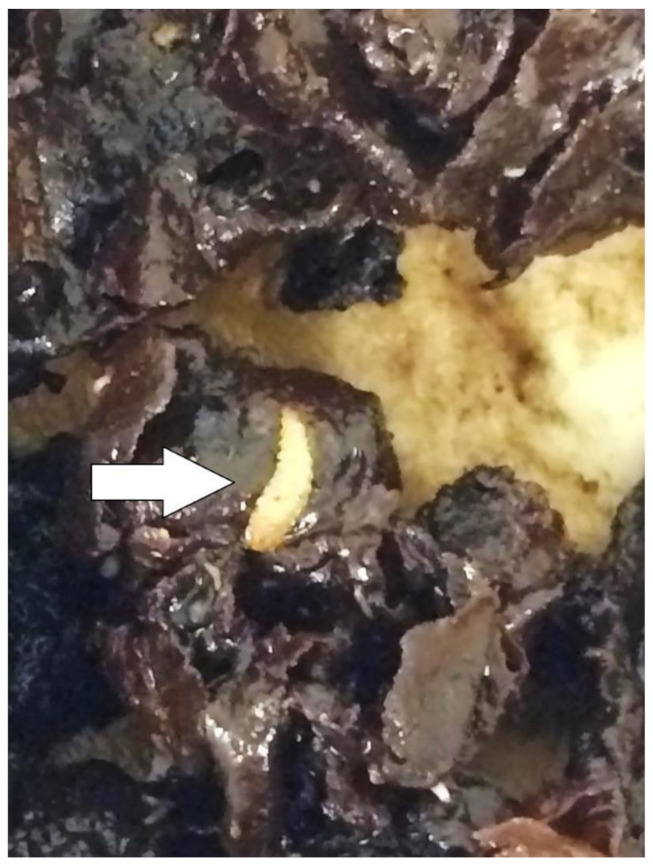
Larva of *Hermetia* sp. (white arrow) found on the decaying stingless bee nest of *G. thoracica* in Peninsular Malaysia.

In early 2019, a sample of a “spoiled” (i.e., language used by beekeeper) *G. thoracica* nest (~300 gm) from Tanjong Ipoh, Kuala Pilah, Negeri Sembilan, Peninsular Malaysia (2°44′31″ N, 102°11′14″ E, 98 m a.s.l.) (Figure 1), was shipped to the Institute for Medical Molecular Biotechnology (IMMB), Faculty of Medicine, Universiti Teknologi MARA. This nest was colonized by several dipteran larvae (Figure 2). Two larvae were killed with hot water and preserved in 90% ethanol, while those remaining (*n* = 30) were reared to the adult stage in an insect cage (40 cm × 40 cm × 40 cm), using the remaining honey and decomposing nest as the food source, in the laboratory environment at 22 °C and 60–70% RH. Larvae were kept inside a plastic container with a wet paper towel fitted on top which was wetted *ad libidum* with water to maintain the ambient humidity. The resulting adults were then removed and euthanized with ethyl acetate. The species identification for adults and larvae was carried out by both morphological and molecular techniques (i.e., sequencing the barcode gene COI).

For molecular identification, two emerged adults were subjected to DNA extraction using the i-genomic CTB DNA Extraction Mini Kit (iNtRON Biotechnology Inc., Seongnam, South Korea). Amplification of the cytochrome c oxidase I (COI) gene was performed in a final volume of 25 μL containing 50–100 ng genomic DNA, 12.5 μL of MyTaq Red Mix (BioLine, Australia), and 10 pmol of each forward and reverse primer adopted from [14]. To infer phylogenetic relationships, reference sequences of *Hermetia* species available from the National Center for Biotechnology Information (NCBI) GenBank were retrieved and subjected to Neighbor-Joining analysis using the Kimura 2-parameter method.

The nucleotide sequences generated from this study were deposited in the NCBI GenBank under accession numbers MT433999 for *H. illucens* and MT434000 for *H. fenestrata.* Adult specimens were pinned, while two larvae were killed in hot water and preserved in 90% ethanol and deposited in the Entomology Collection of the Faculty of Medicine, Universiti Teknologi MARA.

## 3. Results

**Figure 3:**
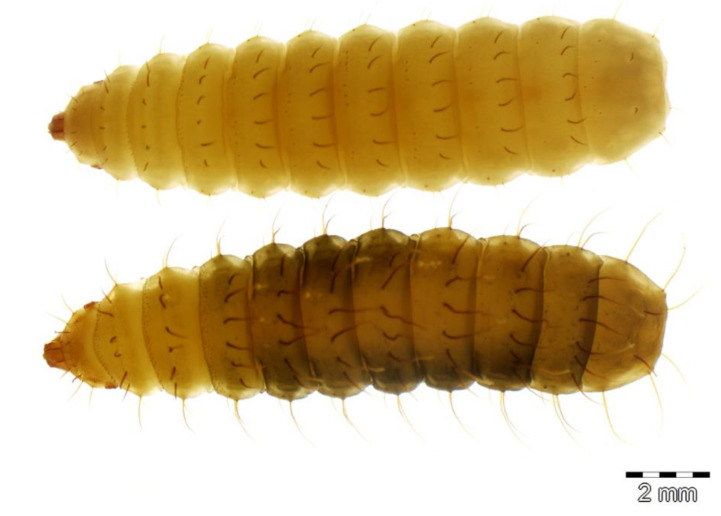
Habitus of the stratiomyid larvae of *Hermetia illucens* (**top**) and *H. fenestrata* (**bottom**) in dorsal view.

**Figure 4:**
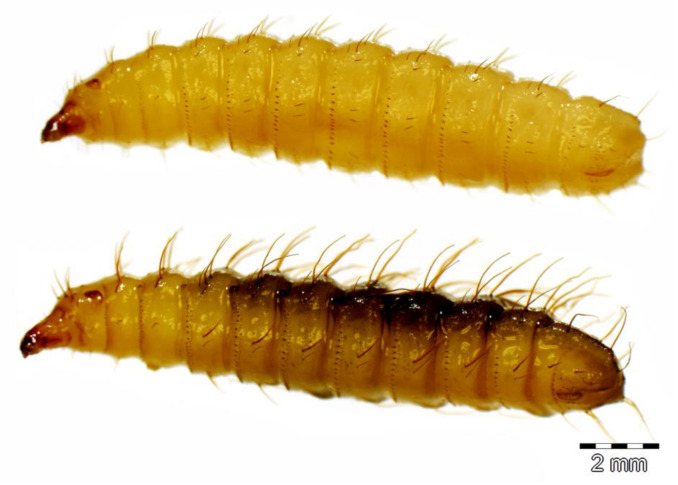
Habitus of the stratiomyid larvae of *Hermetia illucens* (**top**) and *H. fenestrata* (**bottom**) in lateral view.

**Figure 5:**
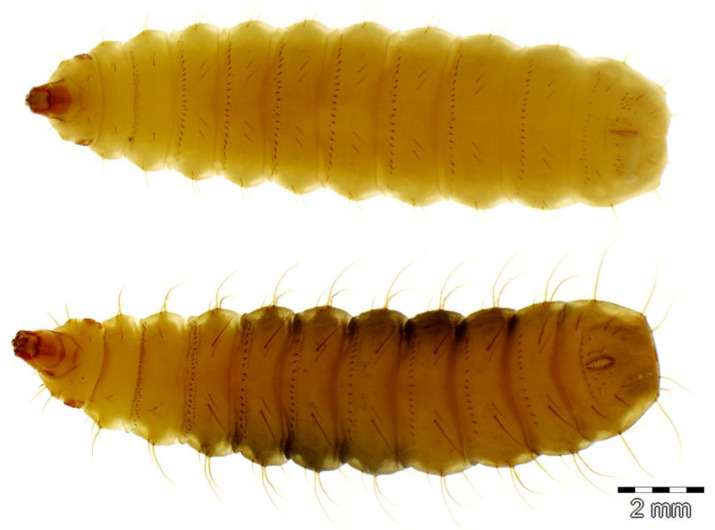
Habitus of the stratiomyid larvae of *Hermetia illucens* (**top**) and *H. fenestrata* (**bottom**) in ventral view.

**Figure 6:**
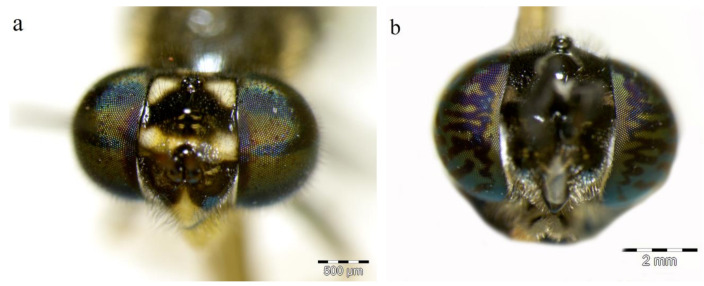
Frontal view of compound eyes: (**a**) Hairy compound eyes of *H. fenestrata*; (**b**) Bare compound eyes of *H. illucens*.

**Figure 7:**
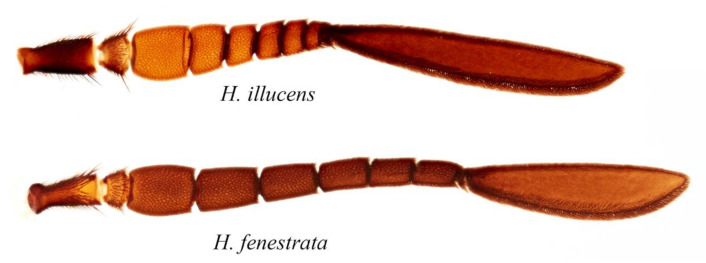
Detail of the female antennae of the adult *Hermetia illucens* and *H. fenestrata*.

**Figure 8:**
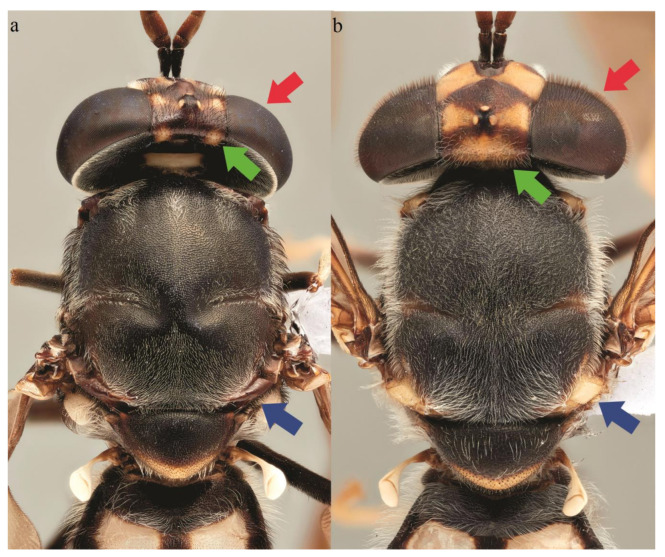
Detail of the thorax [green arrow (median occipital sclerite); blue arrow (postalar callus)] and head [red arrow (compound eyes)] of the adult (**a**) *Hermetia illucens* and (**b**) *H. fenestrata.*

Two stratiomyid species were identified from the decomposing *G. thoracica* nest: *Hermetia illucens* (*n* = 4) and *H. fenestrata* (*n* = 26). Among these larvae, eight *Hermetia* larvae were collected from the substrate provided and the development data and the duration of pupal stage of these samples are presented in Table 1. Puparial development ranged from 2 to 15 days. The larvae of both species resemble each other but differ in some details (Figure 3, Figure 4 and Figure 5). The dorsal setae in *H. fenestrata* are relatively longer than those of *H. illucens* (Figure 3); ventral setae in *H. fenestrata* are relatively more abundant than *H. illucens* (Figure 4). The adults of *H. fenestrata* and *H. illucens* look superficially similar, but they differed in several aspects (Figure 6, Figure 7 and Figure 8). The main difference between both species is that *H. fenestrata* has setose eyes, while *H. illucens* has bare eyes, and while (when alive) *H. fenestrata* has some band-like patterns on the eyes, *H. illucens* has a pattern consisting of short lines with an irregular outline (Figure 6). Also, the proportions of the antennal segments are different: The eighth antennal flagellomere in female *H. illucens* is 1.4 times (*n* = 13, range: 1.3–1.6) longer than all the previous seven flagellomeres together, while in *H. fenestrata* it is shorter, 0.8 times the length of the basal seven flagellomeres (*n* = 11, range: 0.7–0.95). The males of *H. illucens* have the last flagellomere 1.9 times as long as the basal flagellomere (*n* = 6, range 1.5–2.2) and in *H. fenestrata* it is 1.2 (*n* = 3, range 1.05–1.3) (Figure 7). Regarding the thorax and the head of the two species, the postalar callus is yellow in *H. fenestrata* and black in *H. illucens*; the median occipital sclerite has two lateral spots in *H. illucens* and a large central yellow spot in *H. fenestrata* (Figure 8).

**Figure 9:**
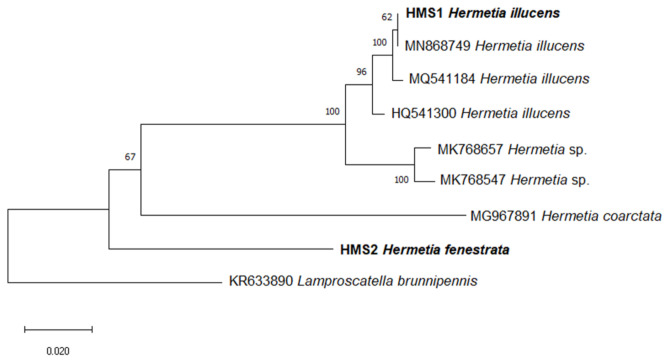
Neighbor-Joining tree of *Hermetia* taxa based on COI sequences. Bootstrap values are shown on the branches. The scale bar represents 0.020 substitutions per nucleotide position. Sequences generated from the present study are in bold.

The specimens identified as *H. illucens* were also confirmed by molecular analysis. The results showed a monophyletic clade with the reference sequences of *H. illucens* (96% bootstrap support). This study also deposited for the first time the sequence of *H. fenestrata* in the NCBI GenBank. *Hermetia fenestrata* was distinctly separated from *H. coarctata* Macquart, 1846, *H.* sp. and *H. illucens* by 16.8%, 16.2–16.3%, and 14.6–15.8%, respectively (Figure 9). These are very high percentage for species in a genus. However, they are consistent with the great genetic difference between the different species of *Hermetia* and also with the difference among *H. illucens* specimens.

## 4. Discussion

There is no key to the Asian species of *Hermetia* and a revision is needed to make sense of the names and species. About 25 species are described from the Oriental and Australian regions [15,16,17,18], but some are synonyms and a few species are still undescribed. All species in the Oriental region are native except of *H. illucens,* which was accidentally introduced from the Americas. The native species found in the stingless beehives belongs to a complex of species which is rather widespread in Asia. They are found from southern China all through Asia down into northern Australia. The species are very similar in appearance but unfortunately the group has not been revised and, therefore, it is not completely clear how many species are actually involved and how many are synonyms. The following species comprise this complex: *H. inflata* (Walker, 1858), *H. fenestrata* de Meijere, 1904, *H. viresncens* (Enderlein, 1914), *H. pallidipes* Hill, 1919, *H. branchystyla* Yang, Zhang, and Li 2014 [17] and *H. olympiae* Lessard and Woodley 2018 [18]. Because there is molecular and morphological evidence that there are at least two species, likely divided by the Wallace-line, the oldest name for the species west of the line would be *H. fenestrata*. In Woodley [15], *H. fenestrata* is considered a synonym of *H. inflata*, but we here consider it as a valid species. This is why we chose this name and very likely *H. virescens* and *H. branchystyla* are synonyms of it.

In 2016, an infestation of a stingless bee nest by so called *“H. illucens”* was reported in northern peninsular Malaysia [12]. According to Hashim et al. [12], the infestation destroyed 250 stingless bee colonies within one week of “attack”, resulting in huge losses to the bee industry. The authors confirmed that the “invading” species was *H. illucens* and suggested to monitor the spread and the potential threat posed by *H. illucens* on the stingless bee industry in Malaysia. However, based on our findings and understanding of the behavior of *H. illucens*, the terms used such as “attack”, “invading”, and “pest” by Hashim et al. [12] might not reflect the true ecology and behaviors of *H. illucens*. In fact, the adult fly illustrated in Hashim et al. [12] was *H. fenestrata* and not *H. illucens*, as evident from the shorter last flagellomere and the white postalar spots (which are black in *H. illucens*), and the presence of a yellow median occipital sclerite, compared to two lateral yellow spots in *H. illucens*. The observation made by Hashim et al. [12] was more likely due to the decaying bee nests attracted female *Hermetia* to lay eggs on the nest.

Furthermore, Hashim et al. [12] stated that the sour smell of honey produced by stingless bees might have attracted female soldier flies to lay their eggs on the nest or log; in fact, this statement has also indicated that the honey had begun to ferment, possibly due to high moisture enabling yeast to ferment the honey [19]. Consequently, the smell of the fermented honey likely attracted *Hermetia* to oviposit, and the larvae consumed the honey as their food source. In the present study, *Hermetia* larvae were not observed to predate or parasitize larvae or pupae of the bees during the colonization process. Also, *Hermetia* adults are only feeding of water, honeydew, nectar, and pollen and are, like all other members of this family, not attacking other insects as adults.

In this current paper, the discovery of *H. fenestrata* in *G. thoracica* nests is reported. The larvae of both *H. illucens* and *H. fenestrata* can develop in a wide diversity of organic matter, from decomposing manure and meat, to fruits and vegetables [20,21,22], spanning from bananas [23] to human remains [24] and animal dung [25]. Although they can cause accidental myiasis [26,27], neither species have not been reported as obligatory parasites thus far. Most probably, the potential cause for stingless bee nest colonization was due to the poor condition of the colony. The bee nests were decomposing and producing a “sour” smell through yeast and/or bacteria metabolism. For this reason, *Hermetia* species were attracted to the substrate searching for oviposition sites, hence, the presence of *Hermetia* was not due to parasitism on the bee larvae, but merely the colonization of a decomposing resource. The best way of preventing such incidents in the future is daily good farm maintenance, and the regular inspection of the colonies.

*Hermetia fenestrata* and the bee species are both native and, they have adapted to each other and should not suddenly become a threat, like a newly introduced species could. *Hermetia illucens* has been in the region for at least 80 years, and there has never been reports of attacks of bee colonies. Now finding both *Hermetia* species together indicates that they are just taking advantage of a rotting food source together, which is a very important observation. There are not many publications on this topic and only a few other instances from Africa and the New World reporting *Hermetia* in stingless and honeybee nests. Schwarz [28] refers to Rau [29], who found a large amount of *Hermetia* larvae in the soil beneath the nest entrance, where the bees dropped their refuse from the nest, and Schwarz [28] speculated that the larvae might have been “original ejections from the nest”, while Rau [29] never mentioned this at all. Nogueira-Neta [30] reported that a Brazilian stingless bee-keeper lost two colonies, which the beekeeper attributed to the presence of *Hermetia*. Although Nogueira-Neta [30] speculated that this colony might have had damaged by water before and concluded that *Hermetia* is more a tenant than an enemy in Meliponini colonies. Copelo [31] reported that *Hermetia* in Argentina is often observed laying eggs in the cracks of European honey bee hives and sometimes develop inside of them, but would not harm healthy hives, and would only be a problem in combination with wax moths. This indicates that *Hermetia* is again not the cause of the hive decline, but just a symptom. There is also a report of *H. illucens* attacking a stingless bee nest in Kenya, Africa [32] where a largely liquefied mass of cells, honey, and larvae were found, but again it is not clear if this is the cause or a symptom for the decline of the colony.

There is an old record of Riley [33] reporting *Apis* honey bee colonies being infested in Alabama, USA, and that the bee keeper observed *H. illucens* laying eggs in cracks of the bee hive, but the larvae which were destroying the colony, which he assumed they were also *Hermetia*, were actually a wax moth according to his description. This was clearly another misinterpretation and *Hermetia* is simply attracted to the decay of the nest and not the cause of the colony destruction.

## 5. Conclusions

In conclusion, the presence of *Hermetia* larvae in decaying stingless bee nests in Peninsular Malaysia is a symptom of a dying colony, or a colony in distress, but it is likely not the cause for the decline and renders new insights and future studies due to its potential economic impact. The frequency of *Hermetia* colonization and its correlation with decomposing bee nests, and the lack of information of the events leading to initial colonization warrants further investigations. In addition, the implementation of proper management of stingless bee farms and novel strategies in prevention and control of *Hermetia* colonization on decomposing stingless bee nests should be further explored in order to maintain and sustain healthy bee colonies. Stratiomyid larvae collected from stingless bee colonies should be properly identified to determine appropriate courses of action for mitigating losses.

## Figures and Tables

**Table 1:** Development data of *Hermetia illucens* and *H. fenestrata* found in stingless bees *Geniotrigona thoracica* nest collected from Negeri Sembilan, Peninsular Malaysia. Larvae and pupae were reared under laboratory conditions at 22 °C and 60–70% RH.

Sample	Initial Date of Specimen Received	Pupariation Date	Pupal Duration (day)	Eclosion Date	Species Identified(Adult Stage)	Sex Identified(Adult Stage)
Larva 1	25.I.2019	06.III.2019	11	17.III.2019	*Hermetia illucens*	Male
Larva 2	25.I.2019	20.III.2019	15	04.IV.2019	*Hermetia fenestrata*	Male
Larva 3	25.I.2019	01.IV.2019	11	12.IV.2019	*Hermetia fenestrata*	Unidentified
Larva 4	25.I.2019	05.IV.2019	10	15.IV.2019	*Hermetia illucens*	Male
Larva 5	25.I.2019	12.IV.2019	4	16.IV.2019	*Hermetia fenestrata*	Male
Larva 6	25.I.2019	12.IV.2019	10	22.IV.2019	*Hermetia fenestrata*	Male
Larva 7	25.I.2019	16.IV.2019	2	18.IV.2019	*Hermetia illucens*	Male
Larva 8	25.I.2019	22.IV.2019	2	24.IV.2019	*Hermetia illucens*	Male
